# Macrophage migration inhibitory factor -173 G>C single nucleotide polymorphism and its association with active pulmonary tuberculosis

**DOI:** 10.1371/journal.pone.0234565

**Published:** 2020-06-11

**Authors:** Mirela Gehlen, Elis Regina Dalla Costa, Maria Lucia Rosa Rossetti, Denise Rossato Silva

**Affiliations:** 1 Programa de Pós-Graduação em Ciências Pneumológicas, Universidade Federal do Rio Grande do Sul (UFRGS), Porto Alegre, RS, Brazil; 2 Centro de Desenvolvimento Científico e Tecnológico, Secretaria Estadual da Saúde do Rio Grande do Sul (CDCT/SES), Porto Alegre, RS, Brazil; 3 Programa de Pós-Graduação em Biologia Molecular e Celular Aplicada a Saúde (Biosaude), Universidade Luterana do Brasil (ULBRA), Canoas, RS, Brazil; 4 Faculdade de Medicina, Universidade Federal do Rio Grande do Sul (UFRGS), Porto Alegre, RS, Brazil; Rutgers Biomedical and Health Sciences, UNITED STATES

## Abstract

**Purpose:**

The establishment of candidate genes associated with susceptibility to TB is a challenge especially due to divergent frequencies among different populations. The objective of this study was to evaluate the association between macrophage migration inhibitory factor (MIF) -173 G>C single nucleotide polymorphism (SNP) and susceptibility to pulmonary TB in a population of southern Brazil.

**Methods:**

Case-control study. Patients > 18 years old, diagnosed with pulmonary TB were included. The control group consisted of blood donors and household contacts, not relatives, healthy and > 18 years old. MIF -173 G>C SNPs were genotyped using real-time PCR using a TaqMan SNP Genotyping assay.

**Results:**

174 patients and 166 controls were included. There were no statistically significant differences between cases and controls regarding genotype prevalence (p>0.05). Comparing patients with normal genotype (GG) with those with at least one C allele, there was also no statistically significant difference (p = 0.135). Also, there was no statistically significant difference comparing the homozygous for the mutation (CC) with the other patients (GG and CG) (p = 0.864).

**Conclusions:**

We did not find association between MIF -173 G>C polymorphism and susceptibility to pulmonary TB.

## Introduction

The natural history of tuberculosis (TB) follows a variable course after the initial infection, with only 10% of infected individuals actually developing clinical disease. Genetic, environmental and bacterial virulence factors can influence the clinical presentation of TB [1]. Many studies indicate that genetic factors play a major role in determining the susceptibility and resistance to TB [2–5].

Polymorphisms of genes play an important role in the occurrence and development of TB. In recent years, many TB susceptibility genes have been detected [6–13]. The macrophage migration inhibitory factor (MIF) is a cytokine with proinflammatory chemokine-like functions that have been recognized to play a central role in mediating a wide variety of immune responses against invading pathogens, and may be associated with the onset and / or progression of TB. In a fact, a metanalysis revealed a strong association of a MIF polymorphism with autoimmune and infectious diseases [14]. In TB, MIF is probably the first cytokine to appear in inflammatory response, inhibits macrophage migration, and promotes macrophage accumulation and T lymphocytes activation in inflamed TB lesions [15,16]. Four polymorphisms were identified in the MIF gene; however, only the MIF-173 and MIF (CATT_5-8_) are related to changes on MIF levels [17]. A few studies had evaluated these polymorphisms in TB, in different populations, with controversial results [16,18–22].

The establishment of candidate genes associated with susceptibility to TB is a challenge especially due to divergent frequencies among different populations [23]. Therefore, studies on the relationship between the susceptibility to TB and genetic polymorphisms in various populations and ethnic groups are of great importance. The objective of this study was to evaluate, in a case-control study, the association between MIF -173 G>C single nucleotide polymorphism (SNP) and susceptibility to pulmonary TB in a population of southern Brazil.

## Materials and methods

### Study design and location

This study was a case-control study done on 174 patients and 166 controls and conducted in two hospitals in a city in Southern Brazil with high incidence of TB (80.4 cases/100,000 inhabitants) [24]: Hospital de Clínicas de Porto Alegre, a tertiary hospital, university-affiliated, with 750 beds and about 300 cases of TB treated per year, and Hospital Sanatório Partenon, a referral hospital for TB treatment. The study was conducted between 2016 and 2019. The study was approved by the Research Ethics Committee of Hospital de Clínicas de Porto Alegre (number 16–0599), and all research was in accordance with regulations. All cases and controls signed Informed Consent Form prior to inclusion in the study.

### Patients

We included patients > 18 years old, diagnosed with pulmonary TB, during any time between the diagnosis and completion of treatment. Patients with concomitant extrapulmonary TB were excluded. The control group consisted of blood donors and household contacts, not relatives, healthy and > 18 years old. Cases and controls were negative for human immunodeficiency virus (HIV), and hepatitis B and C virus (HBV and HCV).

### Data collection

Patients were interviewed using a standardized questionnaire. The following data were recorded: demographic data (gender, age, race, years of schooling); presence of symptoms; smoking history; alcohol and drug abuse, and comorbidities. The racial and ethnic composition of Brazilian society is the result of a confluence of people from many different ethnic backgrounds: the original indigenous people, black Africans, Portuguese settlers, and later, European, Arab and Japanese immigrants, as well as other Asian people and from South American countries [25]. We collected data on race and classified patients in white and non-white. The diagnosis of pulmonary TB was accomplished through chest radiography, sputum smear and culture for mycobacteria, and based on consensus criteria [26]. All patients used the same treatment (the standard treatment in Brazil: rifampicin, isoniazid, ethambutol, and pyrazinamide).

### DNA extraction

Whole blood samples were collected in EDTA tubes. Total genomic DNA was isolated from peripheral blood leukocytes by the salting out method [27] and stored at -20°C until analysis.

### MIF Genotyping-173 G> C

The MIF -173 G>C polymorphism (rs755622) was genotyped by real-time polymerase chain reaction (RT-PCR) using a TaqMan SNP Genotyping assay (part number C_2213785_10; Applied Biosystems, Foster City, CA, USA), according to a protocol of Gomez et al [16], using StepOne ™ Real-Time PCR Systems. The PCR was carried out with mixes consisting of 8 ng of genomic DNA, 2.5 ml of Taqman master mix, 0.125 μl of 20 X assay mix and ddH_2_O up to 5 μl of final volume. The amplification protocol used was 50°C for 2 min and initial denaturation at 95°C for 10 min followed by 50 cycles of denaturation at 92ºC for 15 s and annealing/extension at 60°C for 1 min [16].

### Statistical analysis

Data analysis was performed using SPSS 18.0 (Statistical Package for the Social Sciences, Chicago, Illinois). Genotypic frequencies were tested for Hardy-Weinberg using the chi-square test. Frequencies of genotypes and alleles were compared between cases and controls by logistic regression with adjustment for gender and age. Odds ratio (OR) was used as the point estimates of risk and was calculated along with their 95% confidence intervals (95% CI). P value less than 0.05 was considered as statistically significant. Considering data of a previous study [20], with confidence interval of 95% and a power of 80%, at least 83 cases and 83 controls will be needed.

## Results

During the study period, 174 patients and 166 controls met the inclusion criteria and were included in the analysis. The age range was 18–78 years (cases) and 18–61 years (controls). There were 120 (69.0%) males and 54 (31.0%) females among cases and 105 (63.3%) males and 61 (36.7%) females among controls. One hundred and fifteen patients were white (66.1%) and 59 (33.9%) were non-white among cases and 114 (68.7%) were white and 52 (31.3%) were non-white among controls. The prevalence of genotypes GG (normal), GC (heterozygous), and CC (homozygous for mutation) among cases was 94 (54.0%), 72 (41.4%), and 8 (4.6%), respectively. In controls, the frequencies of genotypes GG (normal), GC (heterozygous), and CC (homozygous for mutation) among cases were 103 (62.1%), 56 (33.7%), and 7 (4.2%), respectively. The genotype frequencies were not found to be significantly different from those predicted by the Hardy–Weinberg equilibrium. There were no statistically significant differences between cases and controls regarding genotype prevalence, controlled for age and sex (Table 1).

**Table 1:** Genotype frequency of MIF -137 G/C polymorphism in cases and controls.

Genetic model	Cases, n (%)	Controls, n (%)	OR (95% CI)	P value
Codominant				
GG	94 (54.0)	103 (62.1)	1.00 (reference)	
GC	72 (41.4)	56 (33.7)	0.71 (0.45–1.11)	0.133
CC	8 (4.6)	7 (4.2)	0.79 (0.28–2.29)	0.675
Dominant				
GG	94 (54.0)	103 (62.1)	1.00 (reference)	
GC + CC	80 (46.0)	63 (37.9)	0.72 (0.47–1.11)	0.135
Recessive				
GG + GC	166 (95.4)	159 (95.8)	1.00 (reference)	
CC	8 (4.6)	7 (4.2)	1.09 (0.39–3.09)	0.864
Allele				
C	88 (25.3)	70 (21.1)	-	0.195
G	260 (74.7)	262 (78.9)	-	0.195

Comparing patients with normal genotype (GG) with those with at least one C allele, there was also no statistically significant difference (p = 0.135). In addition, there was no statistically significant difference comparing the homozygous for the mutation (CC) with the other patients (GG and CG) (p = 0.864).

## Discussion

In the present study, we did not find association between MIF -173 G>C polymorphism and susceptibility to pulmonary TB. There were no statistically significant differences between cases and controls regarding genotype prevalence. In addition, there was no statistically significant difference comparing patients with normal genotype (GG) with those with at least one C allele, and comparing the homozygous for the mutation (CC) with the other patients (GG and CG).

There is increasing evidence that host genetic factors are implicated in susceptibility to TB, corroborated by monozygotic and dizygotic twin studies [5,28], genome-wide linkage studies [29–31], and genome-wide association studies [32,33]. Polymorphisms in candidate genes have shown influence on this TB susceptibility. MIF -173 G>C gene polymorphism has been shown to play a role in TB risk in several studies [16,18–22]. MIF gene is located at chromosome22q11·2 and encodes a T-cell-derived cytokine that plays an important role activating macrophage functions, regulating Th1/Th2 balance, and also is involved in delayed-type hypersensitivity reaction [34–36]. MIF is expressed by epithelial cells of the bronchi and alveolar macrophages, which have substantial influence on defense against tubercle bacilli [37]. In a Ugandan cohort, it was showed that genetic low expressers of MIF were 2.4-times more frequently identified among patients with Mycobacterium tuberculosis bacteremia than those without. Also, MIF deficient mice have an inadequate innate immune response and, consequently, are more susceptible to mycobacterial pathology [38].

We did not find differences in MIF -173 G>C genotype prevalence comparing patients with TB and the control group. Gomez et al [16] studied 230 northwestern Colombian patients with pulmonary TB, negative for HIV, and 235 matched healthy individuals, and found on multivariate analysis that MIF -173C allele was associated with disease (odds ratio = 1.64) in a dominant pattern. In a Moroccan population, the authors demonstrated a statistically significant increase of the MIF –173CC homozygote genotype and MIF –173*C allele frequencies in PTB patients compared with healthy controls [21]. Hashemi et al [18], showed in an Iranian population that the MIF -173 G/C polymorphism increased the risk of PTB in codominant (GC vs GG, OR = 1.76) and dominant (GC+CC vs GG, OR = 1.78) tested models. Three studies were conducted in Chinese population. One of them [19] evidenced that distribution of MIF -173 genotypes (GC + CC) was significantly higher in TB cases than in controls, and the frequencies for MIF -173 (GG vs. GC+CC) were statistically significant different comparing total cases of TB, new cases of TB, and retreatment cases of TB to controls, respectively. Yanlin et al [22] demonstrated that the frequency of MIF -173 (GC + CC) was higher in TB patients than in controls (OR = 2.12). Last, Kuai et al [20] found a significant association of MIF -173 C alleles with susceptibility to active TB. Since polymorphisms occur at different frequencies in populations of different ethnicities, the high prevalence of miscegenation in our country may have contributed to these results [25]. In addition, one should also consider the fact that the population of southern Brazil (study setting) has Italian and German descent, and that these polymorphisms have not been demonstrated in these populations, only in Chinese [19,20,22], Moroccan [21], Colombian [16], and Iranian [18] populations. In addition, the sample size could be small to come to a conclusion at this point; however, we calculated a priori sample size, and it is bigger than 3 out of 6 studies conducted on this topic.

One limitation of this study is that we recruited patients from a single region of Brazil, and, as stated above, it is well known that Brazil is an extensive country with a varied ethnic and racial composition. Therefore, this may have prevented us from finding differences in genotype frequency.

This study has been conducted in mixed population, as both cases and control study subjects consists of peoples from various ethnic origin. An elaborated study with a larger sample size is required to bring enough power to analyse the data from different ethnic groups. In addition, we didn’t collect data on body mass index (BMI), and it is well known that low BMI could contribute to TB disease establishment. Also, the patients were not followed-up after the treatment for relapse. Furthermore, we know that a cohort study including patients with MIF polymorphism and evaluating the development of TB would bring better scientific evidence; however, this kind of study is expensive and take a lot of time to get results. Moreover, MIF levels were not quantified; a correlation study between different genotypes and MIF levels in patients and controls might help to explain the association between MIF -173 G>C gene polymorphism and susceptibility/resistance to tuberculosis. In spite of these concerns, this is the first study to evaluate the association between MIF -173 G>C polymorphism and susceptibility to pulmonary TB in Brazil.

In conclusion, in the present study, there were no differences in MIF -173 G>C genotype prevalence when comparing TB cases and controls. Further studies, including patients from various regions of Brazil are necessary to better evaluate the association between MIF -173 G>C polymorphism and susceptibility to pulmonary TB.
